# On the Formation of Black Silicon Features by Plasma-Less Etching of Silicon in Molecular Fluorine Gas

**DOI:** 10.3390/nano10112214

**Published:** 2020-11-06

**Authors:** Bishal Kafle, Ahmed Ismail Ridoy, Eleni Miethig, Laurent Clochard, Edward Duffy, Marc Hofmann, Jochen Rentsch

**Affiliations:** 1 Fraunhofer Institute for Solar Energy Systems (ISE), 79110 Freiburg im Breisgau, Germany; ahmed.ismail.ridoy@ise.fraunhofer.de (A.I.R.); eleni.miethig@gmail.com (E.M.); marc.hofmann@ise.fraunhofer.de (M.H.); jochen.rentsch@ise.fraunhofer.de (J.R.); 2 Nines Photovoltaics, Dublin 24, Ireland; l.clochard@nines-pv.com (L.C.); e.duffy@nines-pv.com (E.D.)

**Keywords:** dry etching, black silicon, photovoltaics

## Abstract

In this paper, we study the plasma-less etching of crystalline silicon (c-Si) by F_2_/N_2_ gas mixture at moderately elevated temperatures. The etching is performed in an inline etching tool, which is specifically developed to lower costs for products needing a high volume manufacturing etching platform such as silicon photovoltaics. Specifically, the current study focuses on developing an effective front-side texturing process on Si(100) wafers. Statistical variation of the tool parameters is performed to achieve high etching rates and low surface reflection of the textured silicon surface. It is observed that the rate and anisotropy of the etching process are strongly defined by the interaction effects between process parameters such as substrate temperature, F_2_ concentration, and process duration. The etching forms features of sub-micron dimensions on c-Si surface. By maintaining the anisotropic nature of etching, weighted surface reflection (*R*_w_) as low as *R*_w_ < 2% in Si(100) is achievable. The lowering of *R*_w_ is mainly due to the formation of deep, density grade nanostructures, so-called black silicon, with lateral dimensions that are smaller to the major wavelength ranges of interest in silicon photovoltaics.

## 1. Introduction

The formation of high aspect ratio and/or large surface area sub-micron structures on silicon is of high interest for several applications, such as photovoltaics, micro-electro-mechanical systems (MEMS), photodetectors, and silicon anodes for lithium-ion batteries [1,2,3,4]. Meanwhile, in silicon photovoltaics, the formation of submicron features on c-Si surface has received increased attention in the field of Si photovoltaics due to its ability to dramatically reduce the surface reflection to a very low value so that the wafer turns ”black” in appearance, a so-called black-silicon (B-Si). Application of such anti-reflective surfaces on single or monocrystalline silicon (mono-c-Si) and multicrystalline silicon (mc-Si) surfaces have shown an improved short-circuit current density (*J*_SC_) of solar cells due to a higher absorption of incident light [1,2,3].

For applications requiring high volume manufacturing such as a photonic detector or a photovoltaic cell, reduction of process costs is inevitable. However, a high cost of ownership (COO) of the vacuum-based etching equipment might make its application in the photovoltaic industry difficult. Other wet-chemical texturing methods such as metal-catalysed chemical etching (MCCE) also promise low reflection on both mono c-Si and mc-Si wafers. However, this method has drawbacks, such as the need for multiple processing steps, the use of expensive process materials, cumbersome waste management, and a high likelihood of trace metal particles being present in the Si wafer.

As alternatives to wet-chemical etching processes, plasma-based activation of Fluorine-containing gases like F_2_, XeF_2_, SF_6_, CF_4_, and NF_3_ was widely investigated in the past to perform etching of c-Si for different applications such as photovoltaics, micro-electro-mechanical systems (MEMS), optoelectronic devices, and optical filters [4,5]. Chemical dry etching promises to provide significant economic and technological advantages to both of the abovementioned processes. It is known that gases like F_2_, XeF_2_, and ClF_3_ can etch Si spontaneously, even at room temperatures, without any need of plasma excitations [6,7,8,9]. Typically, a good selectivity towards various masking materials including metals, photo-resists, SiO*_x_*, SiN*_x_*, etc.; and an isotropic etching of Si with high etch rates is the most important criterion desired for plasma-less dry etchants in microelectronics and/or MEMS micromachining. In solar cell fabrication, however, the anisotropic nature of the surface roughness left on the Si surface after the etching process is the most important criterion for forming anti-reflective surfaces allowing higher light absorption [10].

In comparison to other spontaneously activated etchants like XeF_2_ and ClF_3_, F_2_ is known to have a lower Si etch rate at room temperature [9]. However, thermal activation of F_2_ gas at moderate temperatures can be used to etch Si wafers with reasonably high etching rates. It has been reported that F_2_ leaves a rougher Si surface than XeF_2_ after the plasma-less etching process at room temperature [6]. However, no detailed knowledge about the surface roughness left after the etching process exists in the literature. In this paper, we study the plasma-less etching of Si in F_2_/N_2_ gas mixture when the Si wafer is heated at moderate temperatures of up to 300 °C. In comparison to other studies on halogen etching of Si, the basis of this study is an industrially available etching tool for products that need a high volume manufacturing platform. We first provide a brief introduction to the experimental tool, followed by a detailed study about the influence of tool process parameters on etch rate, surface morphology, and the resulting surface reflection after the texturing process. The process parameter variation of the etching tool is performed by using design of experiments (DOE) and the resulting output data (etch rate and surface reflection) are analysed using statistical methods. Thereafter, the nucleation of etch pits and the evolution of surface roughness are investigated based on detailed microscopic observations, and activation energies are calculated for the F_2_-Si reaction system. The etching process forms high aspect ratio B-Si features that have potential for different applications; we particularly focus on discussing the properties of nanostructures that qualify them to be used as anti-reflective layers in photovoltaics.

## 2. Materials and Methods

### 2.1. Experimental Tool

**Figure 1:**
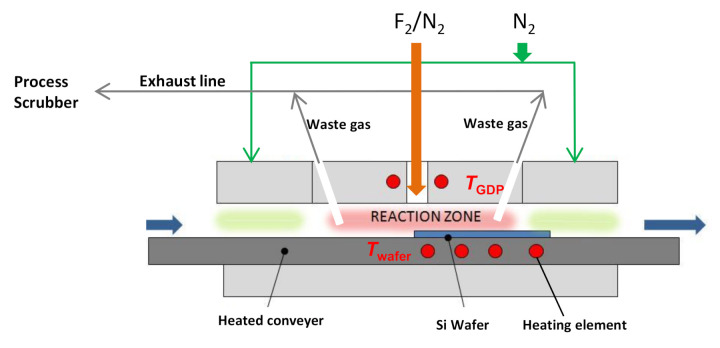
Schematics showing the atmospheric pressure dry etching (ADE) reactor and external connections to gas supply and exhaust lines.

In this work, an atmospheric pressure dry etching (ADE) tool (Nines ADE-100) is used to carry out dry etching of monocrystalline silicon (mono c-Si) and multicrystalline silicon (mc-Si) wafers. The prototype etching tool is manufactured by Nines Photovoltaics and installed at Fraunhofer ISE to establish an etching process that can be easily adapted in high volume production. Figure 1 shows the schematic providing details of the external connections and the reactor of the ADE tool.

All the gas lines are assembled in a valve manifold box (VMB) and mass flow controllers (MFC’s) are used to control the gas flow rates. In the system, F_2_ is the only etching gas present. High purity F_2_/N_2_ mixture in a gas bottle is stored in a gas cabinet. In this experiment, gas bottles filled with F_2_/N_2_ mixture with a maximum F_2_ concentration of 20% are used. The etching is performed in the ADE tool, which is a compact and ventilated enclosure of metal sheets and polycarbonate doors. After the etching process, toxic and corrosive waste gases (F_2_/SiF*_x_*) are removed by the exhaust lines and fed to a dry bed process scrubber (CS clean systems) for abatement purposes. The etching gas is passed through a heated zone (gas diffusion plate GDP) intending to provide a temperature *T*_GDP_ that could potentially facilitate partial dissociation of F_2_ into more reactive F atoms. N_2_ is used as a carrier gas to dilute the effective F_2_ concentration in the F_2_/N_2_ gas mixture during the etching process, as well as a purge gas to purge the gas lines and the reactor chamber after etching. Besides, the reactor is separated from the outer section of ADE tool with the help of two N_2_ gas curtains, which are placed before and after the reaction zone. The gas curtains maintain a continuous flow of N_2_ gas and contain the reactive gases inside the reactor. A slight pressure difference (Δ*P* ≈ 60 Pa) is maintained between the outside and inside of the reactor to contain the leakage of the reactive (both reactant and product) gases released during the etching process. The conveyer system is designed so as to transport the large area Si wafers (15.6 cm × 15.6 cm) through the reactor in an inline mode. The wafers are held in the conveyer system by a minor vacuum (2–3 kPa) and can be heated to a controlled temperature (*T*_wafer_). The wafers are then dynamically transported through the reaction zone and later unloaded on the other side of the conveyer system.

The following nomenclatures of the tool process parameters are used in the paper: (a) flux of F_2_ in F_2_/N_2_ gas mixture: Q_F2_; (b) flux of separate N_2_ as carrier gas: Q_N2_; (c) total gas flux inside the reactor: Q_F2+N2_; (d) effective concentration of F_2_ in total gas flux: σ_F_; (e) set temperature of the gas diffusion plate: *T*_GDP_; (f) set temperature of the wafer substrate holder/heater conveyer: *T*_wafer_; and velocity of the wafer substrate moving through the reaction chamber (*v*).

### 2.2. Design of Experiments

**Figure 2:**
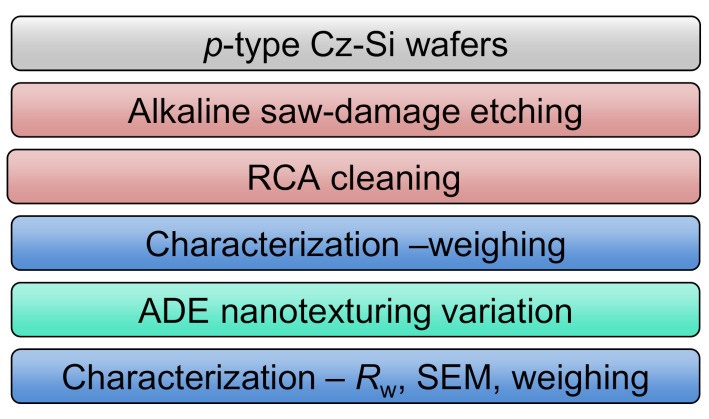
Process flow of the wafers used for the statistical design of experiments.

In order to perform the evaluation of the experimental results in the least biased way, the design of experiments (DOE) is performed using the statistical software. The major process parameters that might have an influence on the etching process are: *T*_GDP_, *T*_wafer_, *v*, Q_F2+N2_, and σ_F_. The total gas flux (Q_F2+N2_) is kept constant, whereas the N_2_ gas flux (Q_N2_) is varied to reach the desired F_2_ concentration (σ_F_) values. Three level factorial design (3 × (*k*−*p*)) with four factors (*k* = 4) and one block (*p* = 1) is used to generate the experimental design. Additionally, one replication is performed in each case, summing up the total number of experiments to be 27 × 2 = 54. Table 1 lists the process parameters as factors that influence the etching process, and Figure 2 shows the workflow of the experiment.

Large area (15.6 × 15.6 cm^2^) *p*-type (100) Cz *c*-Si wafers are first saw-damage etched in alkaline solution and then cleaned using RCA sequence. The wafers are weighed in a weighing scale before transferring them to the etching tool. A variation of process parameters is performed in the etching tool as per the statistical design shown in Table 1. For each set of process parameters, the front side and the rear side of the wafer are etched consequently with the identical process parameters. In the analysis of the data, the etchings of front and rear side are assumed to be replicas of each other. After etching each side, weight measurements are performed to estimate the average value of the etch rate. Additionally, the surface reflectivity is measured in an integrated sphere using a UV/Vis/NIR spectrally resolved spectrophotometer (Varian Cary 5000, Agilent Technologies Germany GmbH, Waldbronn, Germany) in the wavelength spectrum of 250–1200 nm. The weighted surface reflection (*R*_w_) [11] is then calculated in the wavelength spectrum of 300–1200 nm and the weighing function is applied using the internal quantum efficiency of a standard silicon solar cell under AM 1.5 G conditions.

### 2.3. Estimating Activation Energy

**Figure 3:**
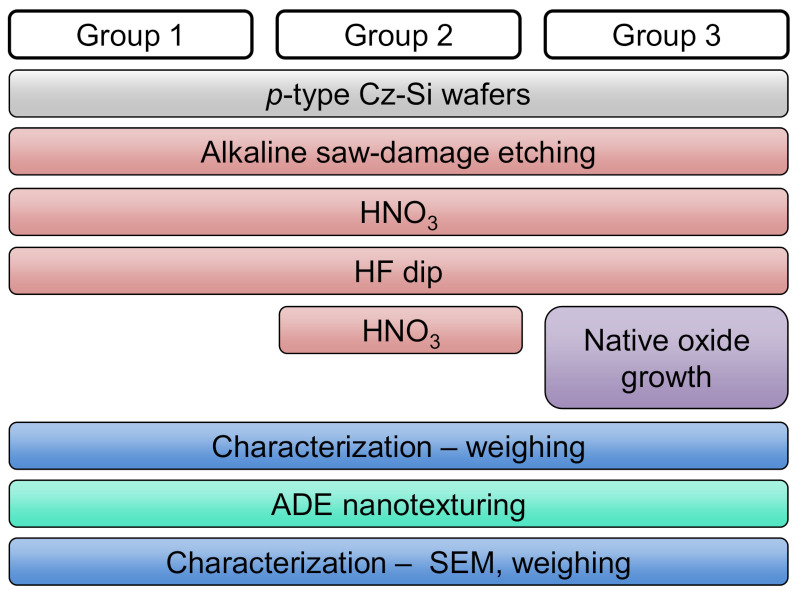
Process plan followed to investigate the Arrhenius behaviour.

To investigate the influence of oxide termination on etching results, the Arrhenius behaviour of etching is investigated. The process plan of the experiment is shown in Figure 3.

The precursors used are *p*-type Cz wafers of (100) crystal orientation after saw-damage etching using alkaline solution. The wafers are then divided into three groups. All groups are cleaned separately using the cleaning sequence of hot HNO_3_ (120 °C, 68%wt, 10 min), HF dip, and DI water rinsing. Afterwards, Group 2 is treated again with hot HNO_3_ solution to grow a homogeneous chemical oxide on Si surface. Group 3 is kept in storage under the exposure of laboratory air for 4 days to grow a native oxide. Please note that the wet-chemical sequences for Group 1 and Group 2 are performed just before the etching process. The wafers from all three groups are etched together in the ADE tool at three different set *T*_wafer_ values. During etching, all other process parameters are kept constant. After the etching, the etch rate is calculated for each group.

The rate of the reaction (here, etch rate) can be expressed in the form of Arrhenius Equation (1)

(1) RSi=k0e−EaRT,

where *R(Si*) represents the etch rate of Si in µm/min, *k*_0_ is the pre-exponential factor in µm/min, *R* is the gas constant, and $E_{a}$ is the activation energy in kCal/mol.

Based on above expression, ln (*R*(*Si*)) is plotted against the inverse of *T* (*T*_wafer_) and $E_{a}$ is calculated.

### 2.4. Characterization of Nanostructures

*p*-type, (100) mono c-Si Cz wafers are first saw damage etched in alkaline solution, and cleaned by RCA cleaning followed by HF dip and DI water rinsing. The wafers are then etched using ADE process. During the ADE process, a variation of etching duration (*v*) is performed, whereas all other parameters are kept constant. Process conditions are chosen (*T*_wafer_ = 200 °C, Q_F2+N2_ = 24 slm, σ_F_ = 5.0%) in order to maintain directional etching in (100) direction, and the directionality of the etching process is verified by SEM measurements (SU 70, Hitachi High-Technologies Corporation, Tokyo, Japan). SEM top-view and cross-sectional view measurements of each sample are performed and five images of each sample are used for the analysis. The dimensions of the nanostructures are extracted by analysing SEM images by using image processing software ImageJ 1.48v [12]. The depth of the nanostructure is extracted simply as the distance from the top of the structure to its valley in the cross-sectional image. In Figure 8i, it was observed that the nanostructure top-view geometry resembles that of an ellipse. With this assumption, the 2D top-view image of the nanostructure is calculated by fitting it as an ellipse. From the measured areas, the diameter of a circular geometry is calculated for simplicity reasons, which represents the lateral dimension or width (*w*) of the nanostructure.

### 2.5. Estimation of Surface Enlargement Factor

The surface enlargement factors (*S*_f_) of textured surfaces are estimated by two methods: (a) atomic force microscopy (AFM, Dimension 3100, Brucker Nano Surfaces (previously Veeco instruments & Digital Instruments, Santa Barbara, CA, USA) with a super sharp tip (Nanosensors SSS-NCH) with tip radius of 2 nm operated in the tapping mode; and (b) the change in weight of wafer after depositing 100 nm atomic layer deposited (ALD) Aluminium oxide (Al_2_O_3_) layer using a spatial ALD tool. The surface area increases with the amount of Si removed during the ADE process. The slope can be well fitted using a linear function (R^2^ = 0.97) for the ALD deposition method and (R^2^ = 0.93) for the AFM method, although a slight discrepancy in calculated *S*_f_ was observed. This is expected due to the inability of the AFM cantilever tip to reach the deep valleys of the nanotexture terrain, thus underestimating the *S*_f_ value.

## 3. Results

### 3.1. Statistical Variation of Process Parameters

The statistical analysis of the experimental section was performed for the etch rate and surface reflection as the dependent variables. It was observed that a normal distribution assumption of the residuals is valid and therefore analysis of variance (ANOVA) was used to analyse the output data.

#### 3.1.1. Analysis of Etch Rate as the Dependent Variable

**Figure 4:**
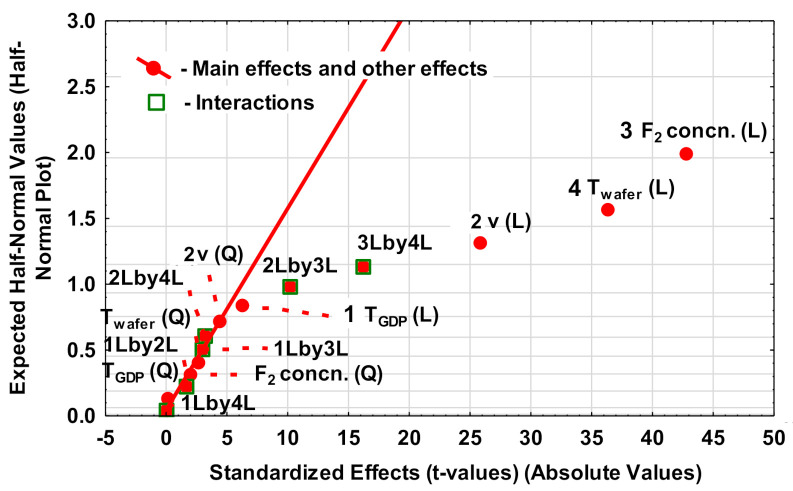
Half-normal plots to identify the significant factors to influence the etch rate. The straight lines in ii) are linear fits of the main effects (red filled circles*)* and interaction effects (green empty squares) that have an absolute value lower than 5. Here, 1, 2, 3, and 4 represent the process parameters *T*_GDP_, *v*, σ_F_, and *T*_wafer_ respectively. L and Q represent the type of effect (linear or quadratic) that the individual process parameters or their mutual interactions have on the dependent variable (here, etch rate). The mutual interaction effects between two parameters, for instance 1 and 2, are represented here as 1L by 2L and 1Q by 2Q for linear and quadratic interactions respectively.

Half-normal plot was used to identify the statistically significant parameters influencing the etch rate and is shown in Figure 4.

This plot is based upon the assumption that all factors that have limited or no effect on responses (here etch rate) fall together, and their estimated effects (either main or interaction) can be fitted very well by a linear function. The outliers have higher statistical significance and the magnitude of the significance increases from left to right. Using this analysis, a large number of interaction effects can be discarded. The main effects of the σ_F_, *T*_wafer_ and *v* are dominant in decreasing order. The linear interactions between σ_F_-*T*_wafer_ and between σ_F_-*v* are less significant. From the half-normal plot, it is observed that the temperature of the gas diffusion plate (*T*_GDP_) is shown to have a very marginal effect on the etch rate. A relatively lower dissociation rate of F_2_ is reported by Steudel et al. (degree of dissociation, α ≈ 4%) at 1000 K [13], and (α < 1%) by Wicke et al. at 600 K [14]. The above reported measurements are performed at the chamber pressure of around 1 bar. Incidentally, this is close to the atmospheric pressure conditions that are used in our experimental set-up as well. Since almost no dissociation of F_2_ is expected for the given experimental conditions, *T*_GDP_ is expected not to influence the etching process.

**Figure 5:**
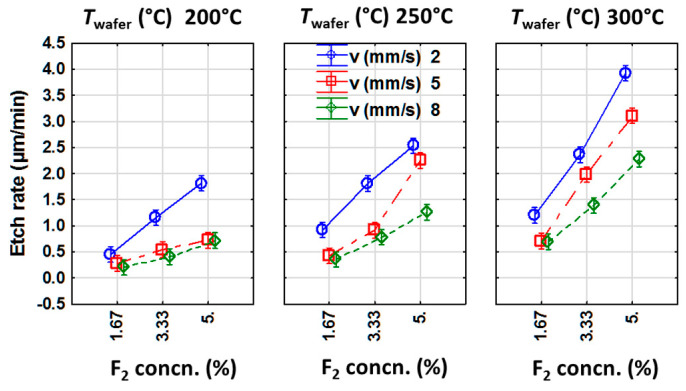
Plot of marginal means and confidence intervals showing interdependency of the most significant factors affecting etch rate. The lines are guides to the eye.

Apart from *T*_GDP_, all other parameters are shown to linearly influence the etch rate in the experimental range of process parameters. Additionally, a linear interaction between σ_F_-*T*_wafer_ and between σ_F_-*v* is observed. These main and interaction effects can be intuitively understood by plotting the marginal mean and confidence intervals, as in Figure 5.

As expected, an almost linear increase in the etch rate is observed for an increasing σ_F_ irrespective of any values of *v* and *T*_wafer_. A decrease in *v* always leads to a higher etch rate, which implies that the etch rate is increasing with the etching duration for each experiment. This is attributed to a possible increase in surface roughness and additionally to an increase in local temperature in the wafer due to the exothermic reaction between F_2_ and Si. In the latter case, the subsequent heat release increases the reaction rate of the newly arriving F_2_ molecules with Si. An increase in σ_F_ leads to a higher availability of F_2_ molecules for the reaction with Si surface. This suggests that the etching process is still limited by the availability of F_2_ in the reaction chamber within the range of process parameters applied in the experiment.

As per the rate equation, a higher temperature of silicon wafer is expected to enhance the etch rate due to an increment in the rate constant of the etching reaction. Here, the influence of increasing *T*_wafer_ on etch rate is marginal for process conditions featuring lowest F_2_ concn. (σ_F_ = 1.67%) and shortest process duration *(v* = 8 mm/s). For an increasing values of σ_F_ and *v*, the influence of *T*_wafer_ on the etch rate increases gradually. Meanwhile, for the combination of longest process duration (*v* = 2 mm/s) and highest fluorine concentration (σ_F_ = 5%), *T*_wafer_ is found to strongly influence the etch rate. For instance, increasing *T*_wafer_ = 200 °C to 300 °C resulted in a two-fold increment of the silicon etch rate at σ_F_ = 5% and *v* = 2 mm/s.

#### 3.1.2. Analysis of *R*_w_ as the Dependent Variable

**Figure 6:**
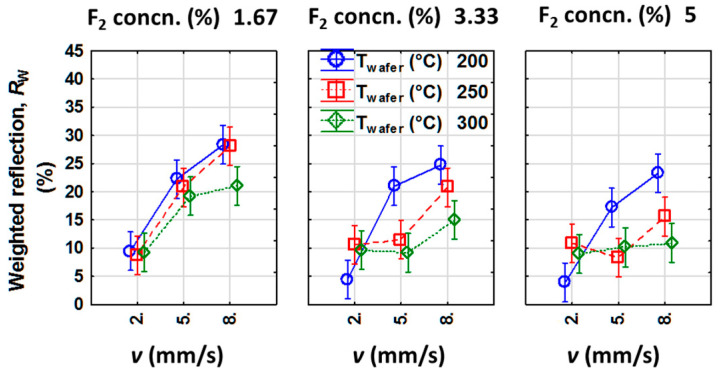
Plot of marginal means and confidence intervals showing interdependency of the most significant factors affecting *R*_w_. The lines are guides to the eye.

From the half-normal plots, *v*, σ_F_, and *T*_wafer_ were identified as the main effects affecting *R*_w_, whereas the interaction effects between *v*-*T*_wafer_, and between *v*-σ_F_ are also dominant. In order to gain more insights about the main and interaction effects, marginal means and confidence intervals of the significant process parameters are plotted in Figure 6.

Please note that for the lowest value of *T*_wafer_ (*T*_wafer_ = 200 °C), the graphs always show the same trend of an increasing *R*_w_ for an increase in *v* irrespective of the σ_F_ used during the etching process. For the *T*_wafer_ = 300 °C, *R*_w_ shows an increasing trend for an increase in *v*; however, only for the lowest σ_F_ = 1.67%. For the highest σ_F_ of 5%, the highest *T*_wafer_ = 300 °C leads to an almost constant *R*_w_ irrespective of the *v* used during the etching process. An optimum (lowest) value of *R*_w_ is achieved for the etching performed with a combination of the lowest *T*_wafer_ (200 °C), the highest σ_F_ (5%) and the lowest *v* (2 mm/s).

#### 3.1.3. Change in Surface Morphology

**Figure 7:**
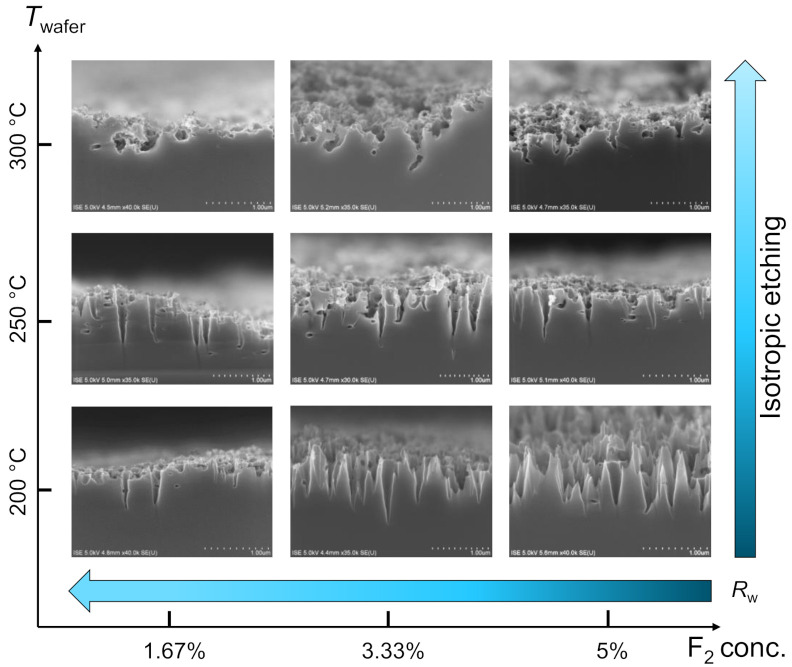
Cross-sectional SEM images of *c*-Si surfaces etched using constant velocity (*v* = 2 mm/s) using different combinations of F_2_ concentration σ_F_ and temperature of the substrate *T*_wafer_. The direction of the arrows represents an increasing value of *R*_w_. The arrows represent an increasing value of *R*_w_ for decreasing σ_F_, whereas an increasing degree of isotropic etching for an increasing *T*_wafer_.

A dramatic change in surface morphology is observed for the change in *T*_wafer_ if the etching is continued for the longest time period (*v* = 2 mm/s), and is summarized in Figure 7. At the lowest temperature (*T*_wafer_ = 200 °C), *R*_w_ gradually decreases for an increasing value of σ_F_ The representative cross-sectional SEM images indicate that anisotropic directional etching towards (100) direction occurs at this particular value of *T*_wafer_ for all values of σ_F_, which results in the formation of conically shaped nanostructures in the c-Si surface.

**Figure 8:**
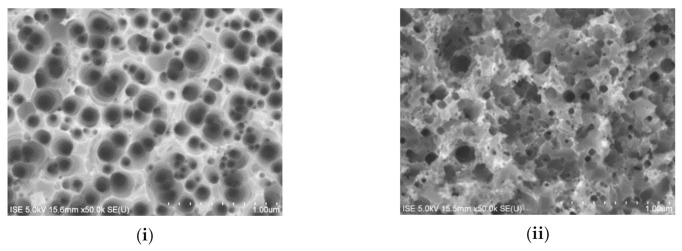
Top-view SEM images of c-Si surfaces etched with σ_F_ = 5% and *v* = 2 mm/s at *T*_wafer_ of (**i**) 200 °C, and (**ii**) 300 °C. The measured weighted surface reflection values are *R*_w_ ≈ 4% (*T*_wafer_ = 200 °C) and *R*_w_ ≈ 9% (*T*_wafer_ = 300 °C).

Here, a decrease in *R*_w_ for an increase in σ_F_ can be attributed to a higher density of nanostructures in the unit wafer area and to an increase in average depth of nanostructures that provides a higher grading of the refractive index from air to Si [15]. As the *T*_wafer_ increases to 250 °C, the directionality of the etching is disturbed and very shallow nanostructures start to form on top of the deeper cone-shaped nanostructures. At an even higher *T*_wafer_ = 300 °C, the deeper cone-shaped nanostructures almost disappear and the c-Si surface consists of only very shallow nanostructures, which, however, do not follow anisotropic etching in (100) direction anymore. The changes in surface structure can be clearly observed in the top-view SEM images of the etched surfaces, which are shown in Figure 8.

**Figure 9:**
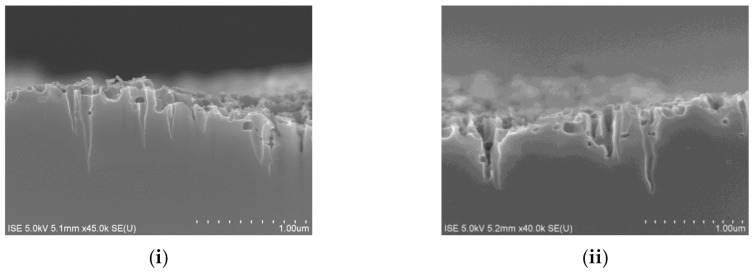
Cross-sectional SEM images showing possibilities of anisotropic etching at (**i**) *T*_wafer_ = 250 °C (σ_F_ = 3.33%, *v* = 5 mm/s) and (**ii**) *T*_wafer_ = 300 °C (σ_F_ = 5%, *v* = 8 mm/s). Both surfaces show an identical *R*_w_ ≈ 11%. The etch rates are measured to be 0.9 µm/min and 2.3 µm/min at *T*_wafer_ = 250 °C and *T*_wafer_ = 300 °C respectively.

The absence of anisotropic cone-shaped nanostructures and the formation of very shallow nanostructures along various crystal planes of c-Si gradually increase the surface reflection. These very shallow nanostructures lead to a “sponge”-like appearance of the c-Si surface. Figure 7 and Figure 8 suggest that an increase in *T*_wafer_ value is mainly dominating the change in surface morphology. However, it is observed that it is possible to compensate the effect of *T*_wafer_ by tailoring the values of *v* and σ_F_. Cross-sectional SEM images of the surfaces that are etched at higher temperatures (*T*_wafer_ = 250 °C and *T*_wafer_ = 300 °C), which, however, still show etching in (100), are shown in Figure 9.

These images provide a qualitative indication that the directional etching property can be maintained to a certain extent even at higher temperatures if F_2_ availability and the duration of etching are controlled. This will be discussed in Section 4.

### 3.2. Initiation of F_2_-Si Etching

**Figure 10:**
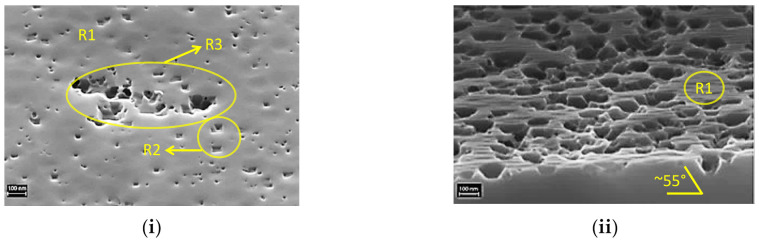
SEM images showing etch initiations in freshly cleaned Si(100) surface after etching with a velocity of (**i**) 6.5 mm/s, and (**ii**) 3.5 mm/s.

Microscopic observations of F_2_ etched c-Si surfaces, which were subjected to HF dip and DI-water rinse before performing the etching process, are used here to comment about the initiation of the F_2_-Si etching process. Figure 10i presents the representative SEM images showing etch initiations in c-Si surfaces etched for different durations. It can be observed that there are three distinct areas in this image: (i) region R1 with no observable etching, (ii) region R2 with small etch pits, and (iii) region R3 with a more vigorous etching and slightly larger and deeper etch pits.

From the first observations, the initial etching seems to start locally at certain locations that feature potentially higher local etch rates than others. Looking at R2 and R3, it becomes obvious that either the nucleation of pits and/or the very initial phases of their propagation show an anisotropic nature in Si(100). The preferential onset of etching for certain locations could be related to the formation of non-homogeneous native oxide during the waiting time between DI-water rinse and F_2_-Si etching process. Although native oxide is reported to have negligible growth until at least 100 min after performing DI-water rinsing [16], heating of the Si wafer with *T*_wafer_ > 170 °C could accelerate the native oxide formation. The abundant pinholes in the oxide layer could provide reaction sites to start the etching reaction. Besides the presence of oxide species, vacancies, defects, and atomic steps are typically known to have a widespread presence in cleaned Si(100) surfaces [17]. Meanwhile, a local increase in roughness of the Si surface during the preparation of wafers for etching (RCA cleaning [18], HNO_3_/HF based cleaning [19], saw-damage etching) can also promote etching by providing reaction sites. An account of F atoms adhering selectively at the reaction sites was reported previously for the HF solution treated Si(100) surface [19].

The anisotropic behaviour of the initial etching becomes more pronounced in the Figure 10ii as the inverted pyramid-like structures are clearly distinguishable. Additionally, a characteristic angle of ≈55 degrees between (100) and (111) crystal planes is observed that indicates that the initial F_2_-Si etching is anisotropic in nature. This is expected for the F_2_-Si etching system because of its sole chemical nature. Anisotropic etching is a known phenomenon typically observed during the etching of Si by alkaline solutions such as KOH, NaOH, TMAH, etc., and is due to the lowest density of surface atoms in (100) among all crystal planes. The side-walls evolve in (111) plane, which is the slowest etching plane due to a much higher density of Si-Si atoms.

### 3.3. Influence of Surface Termination on Activation Energy

Table 2 compares the activation energies of the F_2_-Si etching process measured in current investigations to the ones that are previously reported by other authors for F/F_2_ based etching of Si. It is observed that the surface reaction between F_2_ and Si shows Arrhenius behaviour with a negative slope for an increasing temperature in all cases. This underlines the fact that the F_2_-Si etching reaction is strongly dependent on surface temperature, and suggests that the reaction rate is limited by surface reaction kinetics. It is observed that $E_{a}$ is lowest for the freshly cleaned wafer, slightly increases for the Si surface with native oxide, whereas it is almost twice as high when chemical oxide is grown. Meanwhile, $E_{a}$ calculated for the freshly cleaned Si(100) wafer in this experiment ($E_{a}$ = 12.90 ± 0.13 kCal/mol) is found to be almost 40% higher than the ones reported by Mucha et al. [20] and Chen et al. [21], which is justified by the use of high vacuum in their etching apparatus. Furthermore, F atoms reportedly have a significantly lower $E_{a}$ [22]. Meanwhile, one should be extremely cautious to conclude the influence of temperature on the reaction mechanism just based on these ”apparent” $E_{a}$ values. This is because the formation and the decomposition of SiF*_x_* layer is reported to be temperature dependent and their properties also govern the etch rate [23].

## 4. Discussion

### 4.1. Etching Mechanism

**Figure 11:**
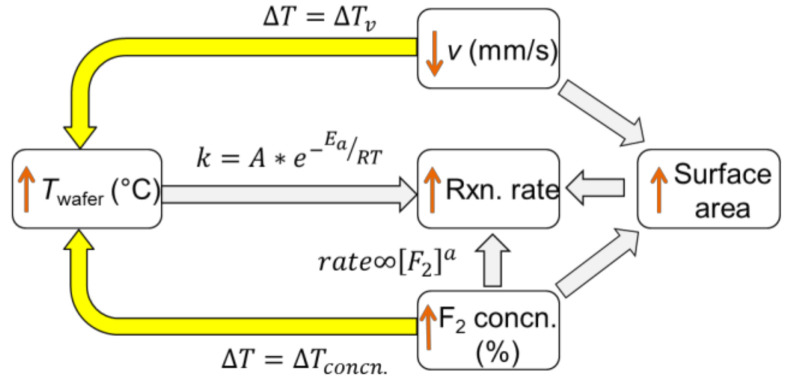
Schematics showing the influence of increasing concentration of the reactant (F_2_) and increasing process duration (lowering *v*) on the resulting temperature and reaction rate of the exothermic reaction system we are analysing here. Here, the temperature dependence of the rate constant (*k*) is shown by the Arrhenius equation with *A* as pre-exponential factor, *E*_a_ as activation energy, and R as the ideal gas constant.

It is observed that the main effects of σ_F_, *T*_wafer_, and *v* mainly determine the etch rate. An increasing etch rate is obtained for a decreasing *v*, an increasing *T*_wafer_, and an increasing σ_F_. In addition, interaction effects are found to be marginally significant. The mutual interaction of parameters can be understood using simple schematic in Figure 11, which shows the dependency of the reaction rate on different process parameters. An increase in etch rate for a decreasing *v* is expected to be a cumulative effect of a subsequent increase in surface roughness, and an increase in surface temperature $∆T_{v}$ due to the exothermic reaction between F_2_ and Si. An increase in σ_F_ increases the reaction rate as per the rate equation. Simultaneously, it also increases the *T*_wafer_ due to the additional heat released $∆T_{concn.}$ as a result of an increased etch rate. A higher value of *T*_wafer_ increases the rate constant (*k*), and thereby the etch rate.

At a particular time *t* after the onset of the chemical reaction, for the case that the set temperature of the wafer substrate holder (*T*_wafer_) is kept constant but σ_F_ and the etching duration is increased, the effective local temperature of the Si wafer (*T*_Si_) can be defined as:

(2) $$T_{Si}=T_{wafer}+∆T_{v}+∆T_{concn.},$$

where *T*_wafer_ represents the initial set temperature of the wafer, and $∆T_{v}+∆T_{concn.}$ represents the increase in wafer temperature due to the heat released depending on the duration of the etching process that also featured an increase in σ_F_. Based upon experimental observations, it can be asserted that the absolute value of *T*_wafer_ is much higher than the factor $∆T_{v}+∆T_{concn.}$ within the experimental range of process parameters.

**Figure 12:**
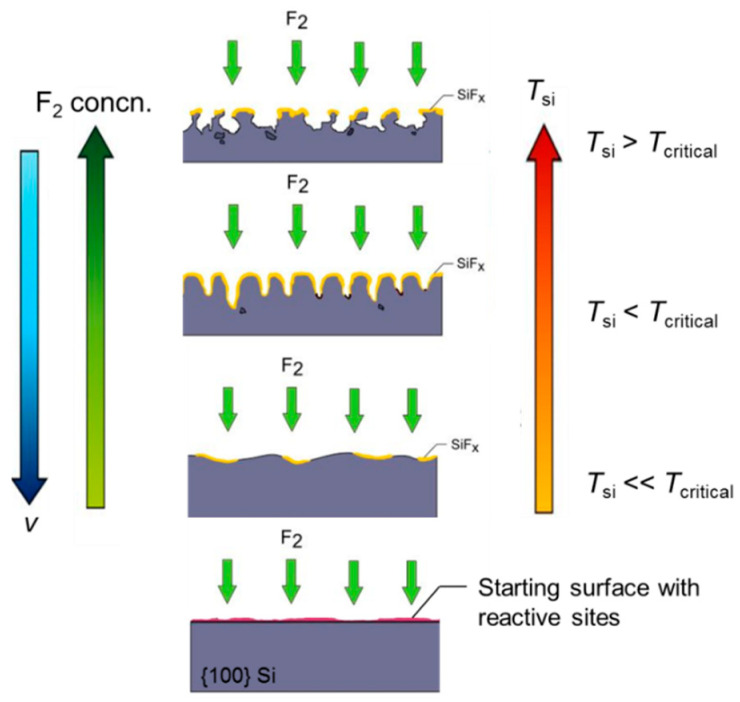
Schematic model of the etching process to explain anisotropic and isotropic etching of Si with F_2_ depending upon the local Si surface temperatures (*T*_Si_).

Based on the results and above discussion, a schematic model of the etching process is presented in Figure 12.

The F_2_-Si reaction is expected to start initially at the reactive sites present in the starting c-Si surface, which is freshly cleaned (H-terminated). The reactive sites could be present due to (a) masking of Si by oxide islands, (b) inherent atomic-scale defects (defects, vacancies, steps) in the surface, and (c) evolution of very fine roughness from the preceding cleaning processes that included oxidizing agents. The differences in local etch rates lead to the nucleation of etch pits. Although the presence of native oxide islands definitely leads to a micro-masking and adds to the inhomogeneous etching behaviour of wafer locations in micron- and nano-scale, the preferential etching behaviour of F_2_ already starts in the atomic scale and is proven by the STM measurements of Nakayama and Weaver [24]. Therefore, it is expected to be the major driving force in the nucleation of pits. It is proposed that the effective temperature of the wafer (*T*_Si_) mainly determines the anisotropic nature of the etching in our experimental conditions. *T*_Si_ is a function of set wafer temperature (*T*_wafer_) and the temperature increase (Δ*T*). The latter is the combination of the heat release during the F_2_-Si etching process for the particular velocity (Δ*T*_v_) and σ_F_ (Δ*T*_concn._). According to Figure 11, the change in σ_F_ and v directly influences the resulting local temperature of the wafer. If the effective local temperature (*T*_Si_) is less than a certain value, an anisotropic and directional etching of c-Si occurs. We call this value of *T*_Si_ as *T*_critical_.

The initial etching is crystal-orientation dependent and leads to the formation of anisotropic features preferably in (100). An increase in surface temperature simultaneously increases the kinetic energy (K.E.) of the adsorbed F_2_ molecules. This leads to an easier surface diffusion of the ad-atoms and allows them to relocate and bind to the reactive sites in the Si surface. This would lead to a faster etching. Furthermore, a higher surface temperature increases the fraction of molecules that have K.E. larger than the required activation energy to proceed with the reaction. This leads to higher etch rates in all crystal planes and an increase in the isotropic nature of etching. Additionally, the rate of formation of product species and its subsequent desorption from the Si surface also increases with an increase in surface temperature. An account of an increasing desorption probability of SiF*_x_* species at higher temperatures is previously discussed by Winters and Coburn [23]. These product species are likely to behave as micro-masks on the Si wafer surface and their degradation with the temperature frees the reactive sites to the incident F_2_ molecules. Hence, a more directional and anisotropic etching is to be expected at the lower effective temperature of Si (*T*_Si_ < *T*_critical_), which leads to the formation of density grade nanostructures in (100) direction. The anisotropic etching mechanism holds true as long as the condition *T*_Si_ < *T*_critical_ remains true, after which a competition between the anisotropic and the isotropic etching occurs. For *T*_Si_ > *T*_critical_, isotropic etching is dominant and no deeper density grade structures are formed on the etched c-Si surface.

### 4.2. Nanostructure Properties

**Figure 13:**
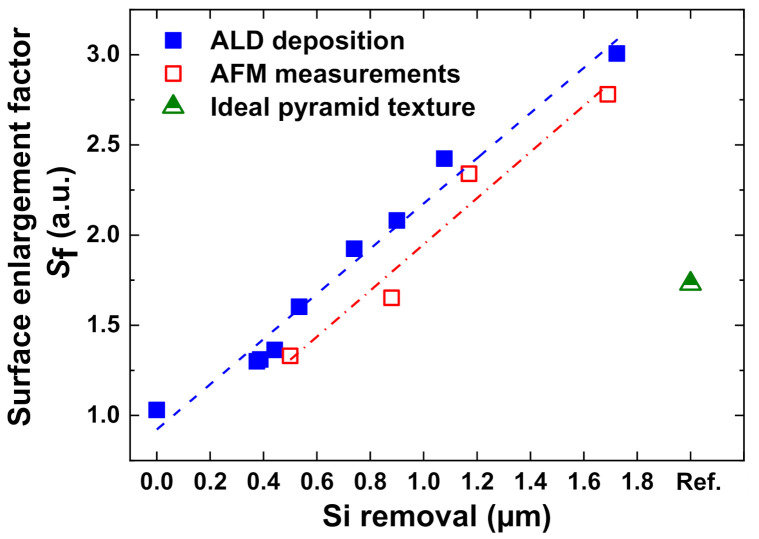
Surface enlargement factor (*S*_f_) of textured surfaces estimated by using atomic layer deposited (ALD) deposition method and by atomic force microscopy (AFM) measurements. The dotted lines represent the linear fit to the data values. For comparison, *S*_f_ of an ideal alkaline (pyramid) texture is also plotted.

Obviously, for Si(100), process parameters should be chosen to maintain a directional etching, which allows formation of deep density graded nanostructures and lower *R*_W_. By maintaining these conditions, it is observed in SEM investigations that the microscopic etch pits progresses into nanostructures with definite geometrical shapes. Figure 13 plots the surface enlargement factor *S*_f_ of the etched surfaces formed at different stages of etching. Here, process duration is varied to achieve various Si removal during etching, whereas all other process parameters (*T*_wafer_ = 200 °C, Q_F2+N2_ = 24 slm, σ_F_ = 5.0%) are kept constant. The process parameter combinations are chosen to ensure directional etching in (100), which is verified by using SEM investigations.

**Figure 14:**
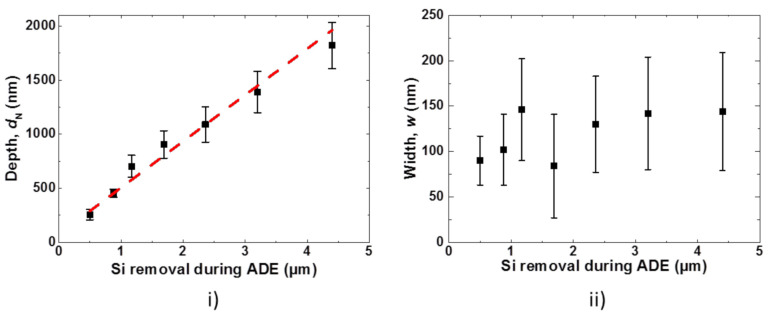
Plots showing change in (**i**) depth (*d*_N_) and (**ii**) width (*w*) of nanostructures for an increasing amount of Si removal during ADE process.

Here, *S*_f_ of the planar wafer (Si removal = 0 µm) wafer is measured to be 1.03. Meanwhile, *S*_f_ increases almost linearly with increasing Si removal during the etching process, leading *S*_f_ ~3.0 at 1.7 µm of Si removal. Figure 14 plots the extracted dimensions of the nanostructures formed after a different amount of Si removal.

An increasing removal of Si, the mean value of nanostructure depth (*d*_N_) increases dramatically from 260 nm to up to 1822 nm. In the case of nanostructure width (*w*), an increase in the mean value of *w* is not clearly distinguishable due to a large standard deviation associated with the estimated data. Therefore, the influence of an increasing period of nanostructure on the surface reflection value is considered here as non-significant. Nevertheless, it should be noted that the maximum value of *w* is smaller than the wavelengths (*λ*_light_) that are most important for Si photovoltaics (400–1000 nm). Under conditions of *w* ≤ *λ*_light_, the lowering of surface reflection occurs either due to the formation of effective medium (*w* << *λ*_light_) and/or diffraction optics (*w* ≈ *λ*_light_).

**Figure 15:**
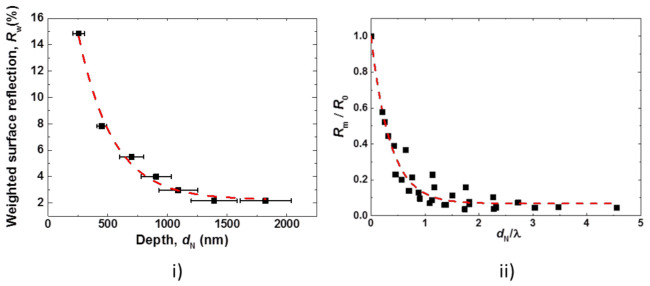
Plots showing (**i**) exponential reduction of normalized reflection for an increasing depth of nanostructures, and (**ii**) *R*_m_/*R*_0_ at each value of *d*_N_/*λ* of nanotextured surfaces.

In Figure 15i, measured weighted surface reflection (*R*_w_) values are plotted against the estimated depth of nanostructures (*d*_N_). *R*_w_ decreases gradually with an increasing value of *d*_N_ and the trend can be very well fitted by an exponential decay function (R^2^ = 0.98). Meanwhile, the saturation of *R*_w_ occurs once the depth of the nanostructures exceeds a certain value. For instance, the weighted surface reflection value falls to *R*_w_ ≈ 5% for *d*_N_ ≈ 700 nm and to a low value of *R*_w_ ≈ 2% for *d*_N_ ≈ 1100 nm in the case of ADE nanotextured surfaces. In Figure 15ii, the normalized reflection value is plotted against the ratio of depth to wavelength (*d*_N_/λ). The normalization of the reflection value (*R*_m_) at each wavelength is performed with the measured reflection value of a saw damage-etched planar c-Si surface (*R*_0_).

The influence of the scattering is minimized by not considering the wavelengths lower than 400 nm. Such a dimensionless quantity (*d*_N_/λ) was previously used to explain the influence of nanostructures formed after MCCE on the surface reflection value [15]. The progression of nanostructure geometry is, however, significantly different for ADE texture compared to MCCE texture. Here, a scatter of the data points is observed, which is attributed to the possible systemic errors in extraction of nanostructure dimensions from SEM images. Nevertheless, the reflection *R*_m_ as a function of *d*_N_/*λ* can be very well fitted by an exponential decay function (R^2^ = 0.97).

(3) $$R_{m}\left(d_{N},\;\lambda \right)=R_{0}\left(d_{N},\lambda \right)\left(A1\times \exp\left(-\frac{d_{N}}{\lambda \times t1}\right)+y0\right)$$

The exponential decay fit to the above equation gives y0 = 0.07, A1 = 0.93, and t1 = 0.35. The plot suggests that the reflection of the nanostructured surface decreases to less than 10 times the value of the SDE surface if the depth of the nanostructure is comparable to the wavelength of interest, i.e., *d*_N_/*λ* ≈ 1. It was observed that in our case, the required values of grade depth to reach *R*_m_/*R*_0_ below 5% are higher (*d*_N_/*λ* ≥ 2) than the ones observed [15] for MCCE structures (*d*_N_/*λ* ≥ 1). This is attributed to a higher lateral dimension of ADE nanostructures in comparison to MCCE nanostructures.

## 5. Conclusions

In this paper, an alternative dry etching process is developed for its application in c-Si solar cells. The dry etching process utilizes spontaneous etching of Si by F_2_ gas in atmospheric pressure conditions. The etching processes result in the formation of surface structures with dimensions in the sub-micron range, also known as nanostructures. Etching of Si by F_2_ gas starts anisotropically and inverted pyramid-like structures are observed at the onset of etching. It is observed that the etching begins non-homogeneously in the Si surface. This phenomenon is attributed mainly to an accelerated attack of F_2_ on surface defects and on the surface sites that are free from native oxide islands. It is proposed that the etching conditions result in an effective local surface temperature that is higher than a certain critical temperature, a highly isotropic etching of Si occurs and a ”porous” looking Si surface is formed. In the other case, nanostructures with well-defined geometry and characteristic dimensions in sub-micron range are formed. Process parameters can be varied to reach even lower *R*_w_ values for an increasing Si removal during the etching process. This is correlated to an increase in the characteristic depths of the nanostructures, which dramatically lowers the weighted surface reflection (*R*_w_) of c-Si in the wavelength spectrum of 400–1000 nm, the main range of interest for c-Si solar cells. As a consequence, a low value of weighted surface reflection *R*_w_ ≤ 2% is achievable due to the formation of black silicon-like features.

## Figures and Tables

**Table 1:** Process parameters used for 3 × (*k*−*p*) experimental design.

Process Parameters	Units	Levels		
		Low	Medium	High
***T*_GDP_**	°C	200	245	290
***T*_wafer_**	°C	200	250	300
***σ*_F_**	%	1.67	3.33	5
***v***	mm/s	2	5	8

**Table 2:** Comparison of $E_{a}$ of F_2_-Si etching estimated in this section to those measured by other authors in various conditions. No previous account of estimation of $E_{a}$ is available for F_2_-Si etching in atmospheric pressure (Atm.) conditions.

Etching Species	Starting Surface	$E_{a}$ (kCal/mol)	Reactor Pressure	Reference
F_2_	Si (100) after HF dip	12.9 ± 0.1	Atmospheric	This work
F_2_	Si (100) with native oxide	13.7	Atmospheric	This work
F_2_	Si (100) with HNO_3_ oxide	23.0	Atmospheric	This work
F_2_	Si (100), Si(110), Si (111)	8.0	Vacuum	[16]
F_2_	Si (100)	9.3 ± 1.8	Vacuum	[17]
F	Si (100)	2.5 ± 0.1	Vacuum	[18]
